# Teaching community health assessment through a global health elective in rural Haiti

**DOI:** 10.5116/ijme.58ba.7ea4

**Published:** 2017-03-29

**Authors:** Paul Dassow, Robert Zylstra

**Affiliations:** 1 University of Tennessee at Chattanooga, Department of Family Medicine, USA

**Keywords:** Teaching, community health assessment, CHA, global health elective, rural, Haiti

## Introduction

The concept of community health has been an important part of Family Medicine since its inception.^1^ Families live in communities, and community resources, characteristics, and culture clearly influence health at both the individual and family levels. Learning how to create and execute a Community Health Assessment (CHA) is a valuable skill for primary care providers as it can assist inprioritizing and resolving the current threats to health for a given locale.^2^

Despite the renewed emphasis on promoting medical resident education in public health, a report examining the state of community health education published in 2015 noted that “research/evaluation and population health are areas not taught well.”^3^ The authors concluded that “improvements are needed” and may be supported by “targeted training in rural or suburban areas”. Various tools have been described in the literature for assisting in community health assessment, but these were created to assist hospitals in meeting the relatively new CHA requirement in the Accountable Care Act.^4^ The purpose of this article is to describe the implementation of a curriculum to teach community health assessment during a Global Health elective and the subsequent lessons learned.

### CHA Development

The Department of Family Medicine at the University of Tennessee College of Medicine at Chattanooga initiated a Global Health Elective in June of 2014.  In planning for this annual, month long elective, the Department’s faculty chose to incorporate the development and execution of a community health assessment into the curriculum.  This curriculum was designed as a 4 step process involving faculty, residents and medical students.

Step 1 of the CHA design process was a 90 minute round table meeting of the Global Health team commenced after the team arrived on site, which in 2014 was a village in the mountains of Central Haiti.  This meeting was dedicated to brainstorming about the varied factors that might influence the community’s health, discussions about how this community might view health as a construct, and logistic factors involved such as appropriate assessment length and methods for obtaining the desired information.

Step 2 was a 60 minute round table meeting conducted after 3 days of clinic work to create the actual assessment tool.  The initial 2014 group decided that a house-to-house survey would be the most effective method for obtaining accurate information. A subsequent group opted for a survey delivered to patient’s presenting for a clinic visit.   Step 3 involved the deployment of the assessment tool.  For the 2014 team, a half day was set aside to visit 30 homes by 3 teams, each with its own translator.  The group opting for the clinic-based survey utilized the Department’s behavioral health specialist accompanied by a translator to interview patients waiting for their appointment.

Step 4 of the process involved creating a user friendly report of the collected data, assessing the data for areas of highest need that were amenable to change, and suggesting possible future interventions for subsequent medical teams.

### Lessons learned

#### Lesson #1 – Family Medicine residents “get” the social determinants of health

In our design of a community health assessment, the faculty was particularly interested in what aspects of social health the residents would find pertinent and worth measuring.  We were somewhat surprised by the emphasis that the residents placed on living conditions as well as economic factors in their survey design.

Out of 21 questions included in the assessment tool used in 2014, 11 directly addressed social indicators of health.  These items included size of home, numbers living in home, source of income, family educational attainment, literacy, financial expenditures, and greatest resource needs.  These questions did not include 3 items assessing food and water quality, 1 item regarding spirituality, and 1 item regarding marital status.  The residents argued that these items were all needed to get a complete picture of the health needs of this unique community.

#### Lesson #2 – Home visits are welcomed by families living in low resource environments

One concern the team had about conducting home visits was how these would be received by the Haitians. In the community we were working in, most families live in small huts without indoor plumbing.  Would families be willing to speak to a group of affluent Americans who came knocking on their door asking many personal questions about their life and health?

Each of our 3 teams experienced a gracious reception at all the homes that were visited.  Not only were families eager to tell their story, most wanted to sit on their front porch for longer than we intended and most offered the teams something to eat or drink.  Hospitality clearly extended to strangers, and these people were not afraid to divulge their struggles, family needs, and healthcare concerns.  Residents commented that making these home visits was one of the most educational aspects of their month.

#### Lesson #3 – Evaluating behavioral health issues may be complicated by language and cultural factors

The community health assessment used during our 2015 elective included the 2 question Patient Health Questionnaire (PHQ-2) depression screen. Responses to these questions almost universally addressed functional deficits rather than the symptom patterns we typically encounter in the US. While these responses may have been an accurate representation of our patients’ greatest concerns, we questioned whether our interpreters were able to adequately translate nuances related to emotional health.

From a clinical standpoint, our residents often noted a “flat affect” and the complaint of “no energy” during their patient encounters.  The concept of depression, however, seemed very foreign to this population. Our lack of effective tools (and language) to assess behavioral health resulted in relying on clinician gestalt rather than any specific diagnostic criteria for diagnosing and treating depression.  Residents reported that their understanding of cultural variations in depression presentation was greatly expanded by the experience.

#### Lesson #4 – Data acquisition methods need to be context driven

Medical residents learned that obtaining community health information during home visits offered a contextual richness that can only be inferred from information collected during clinic visits.  Conducting interviews in the clinic waiting area, on the other hand, allows for a more efficient means of data collection. During our 2014 elective, we closed the clinic for a full half day in order for each of our teams to make their 10 home visits. This clinic closure was difficult to explain to those traveling from a distance to access our services. Collecting data from patients as they were waiting to be seen in the clinic was faster, utilized many fewer resources, and allowed for data acquisition from a much larger geographical area.

During our teams’ debrief, we concluded that the best method for data collection will hinge on the types of community health data that are being sought and the teams’ available resources.  Given the difficulty of assessing mental health issues in this context, exploring family dysfunction during home visits will likely yield more accurate data.  Gathering data in a very rural area will likely need to be done in the clinic setting.

## Conclusions

Teaching community health assessment during a global health elective provides medical residents with a rich environment to learn all aspects of the assessment process.  Collected data can be used for empathy building, epidemiological and clinical research, as well as health needs prioritization. Longitudinal tracking of medical residents is planned to determine if training in CHA development and implementation is carried over into their future medical practice.

### Conflicts of Interest

The authors declare that they have no conflict of interest.
